# Tympanocentesis or not? A prospective cohort study for obstructive sleep apnea children in conjunction with asymptomatic otitis media with effusion

**DOI:** 10.3389/fmed.2026.1706426

**Published:** 2026-05-12

**Authors:** Junji Yao, Tingting Jin, Anying Huang, Zhaoyan Wang, Zhihua Zhang, Lingxiang Hu

**Affiliations:** 1 Department of Otolaryngology-Head and Neck Surgery, Shanghai Ninth People’s Hospital, Shanghai Jiao Tong University School of Medicine, Shanghai, China; 2 Ear Institute, Shanghai Jiao Tong University School of Medicine, Shanghai, China; 3 Shanghai Key Laboratory of Translational Medicine on Ear and Nose Diseases, Shanghai, China; 4 Department of Otolaryngology & Head and Neck Surgery, Ruijin Hospital, Shanghai Jiao Tong University School of Medicine, Shanghai, China

**Keywords:** adenoid tonsillectomy, childhood obstructive sleep apnea, childhood otitis media with effusion, observational study, tympanocentesis

## Abstract

This prospective cohort study explored an individualized approach for managing obstructive sleep apnea (OSA) in children with asymptomatic otitis media with effusion (OME). The study compared the outcomes of tympanocentesis and watchful waiting in pediatric patients with OSA and coexisting asymptomatic OME. Pediatric OSA patients (*n* = 73) with asymptomatic OME were enrolled and categorized into tympanocentesis (*n* = 35) and observation (*n* = 38) groups based on parental treatment choice. Adenoidectomy and tonsillectomy were performed for all participants. Audiometric testing, tympanograms, and otoscopy were employed for outcome evaluation during a 1-year follow-up. The tympanocentesis group exhibited immediate and sustained improvements in hearing, with a faster resolution of OME than the observation group. However, Cox regression analysis revealed no significant difference in the overall time to healing between the two groups. Both interventions demonstrated efficacy over the 3-year follow-up period, with minimal recurrence. This study suggests that watchful waiting and tympanocentesis can yield positive outcomes in pediatric patients with OSA and asymptomatic OME. Tympanocentesis may lead to a shorter duration of OME, offering a simplified, optimized, economical, and effective treatment strategy. The findings emphasize the importance of individualized treatment decisions to address each patient’s unique needs.

## 1 Introduction

Both otitis media with effusion (OME) and obstructive sleep apnea (OSA) are common childhood diseases with high prevalence and serious long-term complications. Adenoid hypertrophy (AH) represents a typical causative factor for both OME and OSA, stemming from mechanical or anatomical issues. OSA is common in the pediatric population and affects 8–27% and 1–5% of the pediatric population by simple snoring and OSAS, respectively (1, 2). A previous investigation observed increased knowledge and awareness of obstructive breathing problems among parents, which might improve the early recognition of related symptoms and lead to an elevated likelihood of seeking professional help from clinicians (3). Adenoidectomy and tonsillectomy are the initial and standard treatment methods for pediatric OSA patients. During the surgery evaluation, OME might be found to be a comorbidity. It has been reported that 29.2% of children with adenoid hypertrophy have asymptomatic OME (4). Indeed, it is extremely common in clinical practice that OME is discovered during the planned adenoidal operation.

*Clinical Practice Guideline: Otitis Media with Effusion (Update)* gave a strong recommendation that “Clinicians should manage the child with OME who is not at risk with watchful waiting for 3 months from the date of effusion onset (if known) or 3 months from the date of diagnosis (if onset is unknown)” (5). However, among OSA children complicated with OME, some of them may have obstructive breathing problems but with insignificant symptoms of OME, and OME can be found only during the preoperative examination of planned adenoidectomy for OSA. The guideline also points out if hearing loss is identified, surgery should be performed for OME, including tympanostomy tubes, adenoidectomy, or both (5). For these children with no complaints of OME, whether active treatment should be taken is still a problem. Watchful waiting could be considered as per guidelines, yet the parents might still doubt the possibility of facing a second general anesthesia surgery for OME after 3 months of follow-up.

In our practice, after a detailed consultation about the illness and treatment choices with their surgeons, the parents can choose watchful waiting or active treatment. The purpose of this study was to discuss whether asymptomatic OME requires concurrent treatment in OSA children planned for surgery. Real-world data was collected and analyzed to investigate the prognosis of different interventions (tympanocentesis or watchful waiting) in those children with OSA and asymptomatic OME.

## 2 Materials and methods

### 2.1 Population and enrollment

This prospective cohort study included pediatric patients with OSA and asymptomatic OME at the Department of Otorhinolaryngology of Shanghai Ninth People’s Hospital between January 2019 and April 2021. This study was approved by the Ethics Committee of Shanghai Ninth People’s Hospital. The participants’ guardians signed the informed consent on their behalf.

Inclusion criteria: (1) age 3–12 years; (2) confirmed diagnosis of OSA and admitted for surgical treatment; (3) denial of previous history of OME; (4) diagnosed OME during preoperative examination with both of the following criteria: (i) otoendoscopy: middle ear effusion in one or both ears; (ii) preoperative audiological examination: tympanogram of type B or C in one or both ears, pure tone average (PTA) < 35 dB HL; (5) normal development in height, weight, and speech function; (6) signed informed consent by the parents (or other legal guardians).

Exclusion criteria: (1) previous myringotomy with or without insertion of ventilation tubes; (2) previous adenoidectomy or tonsillectomy; (3) history of ear surgery; (4) cleft palate; (5) Down’s syndrome; (6) congenital malformations of the ear; (7) cholesteatoma or chronic mastoiditis; (8) perforation of the tympanic membrane; (9) PTA ≥ 35 dB HL or other conductive hearing loss attributed to destructive changes in the middle ear, or sensorineural hearing loss.

### 2.2 Audiometric testing

According to the age factor and after individual assessment for each patient, play or standard pure tone audiometry was used to determine hearing threshold levels at frequencies between 500 and 4,000 Hz. OME was considered when conductive hearing loss existed with Type B or C tympanogram, along with visible tympanic fluid under otoscopy.

### 2.3 Grouping and surgical procedures

Since the parents did not previously recognize symptoms related to OME in their child, it was essential to provide them with comprehensive information about the treatment for this newly diagnosed condition. The clinician objectively addressed the following questions in detail. (1) The extent of the child’s current OME, including the amount of fluid in the tympanic cavity and the level of hearing. (2) Whether the child with OME could safely undergo watchful waiting for 3 months from the onset of effusion (if known) or 3 months from the date of diagnosis (if the onset is unknown). If OME persisted after this period, tympanic tube placement was probably necessary, which would require general anesthesia. (3) The potential surgical risks of tympanocentesis, such as secondary infection or perforation of the tympanic membrane.

It was crucial to ensure that the child’s family understood that the child had not yet met the criteria for tympanic tube insertion. The options available were tympanocentesis or observation, each carrying its risks, allowing the family to make an informed treatment decision. The children were classified into a tympanocentesis group and an observation group according to their parents’ choice of treatment for OME. The same treatment for both ears was used for the bilaterally involved children.

Surgical protocol: All children underwent adenoidectomy and tonsillectomy (partial or total) by endoscopic radiofrequency coblator for OSA under general anesthesia, and the morphological assessments of adenoid hypertrophy were recorded. In addition, the children in the tympanocentesis group underwent tympanocentesis through puncture using a straight needle in the inferior-anterior portion of pars tensa followed by aspiration of the effusion. The same surgeon group performed all assessments and procedures.

### 2.4 Follow-up and efficacy evaluation

It was a long-term follow-up study, and at least six postoperative follow-up visits (1 month, 3 months, 6 months, 9 months, 12 months, and 3 years after surgery) were planned. On each follow-up, the examinations included routine ear, nose, and throat checkups, otoscopy, PTA, and tympanogram. OME was considered resolved when hearing normalized to Type A tympanogram, and no tympanic fluid was seen under otoscopy.

The quality of life of the pediatric patients was evaluated. At 1 month, 12 months, and 3 years after surgery, the parents were invited to complete the Otitis Media-6 (OM-6) questionnaire. This assessment evaluates seven aspects: physical pain, hearing loss, speech disorders, emotional disorders, activity limitations, and parental concerns. The scoring scale ranges from 1 to 7 points, with higher scores indicating more severe conditions. The OM-6 is a specialized questionnaire designed to measure the quality of life in children with otitis media and demonstrates high reliability and validity. The researchers in this study established close contact with the parents of the pediatric patients, filling out the case report form accurately and on time. They conducted postoperative follow-ups by notifying them in advance by telephone to ensure that they could attend the follow-ups on time.

### 2.5 Statistical analysis

Statistical analyses were conducted using SPSS Statistics, Version 23 (IBM Corp., Armonk, NY, USA) and Prism Version 9.0 (GraphPad Software, Boston, MA, USA). Mean ± standard deviation or median (range) was used for descriptive statistics of continuous variables with normal or non-normal distribution. Student’s t-test or Mann–Whitney U test was used for inter-group comparison of normal or non-normal distribution. Frequency (proportion) was used for descriptive statistics of categorical variables, and the chi-squared test was used for inter-group comparison. Kaplan–Meier curves were used to describe the efficacy of OME over time, and the log-rank test was used to compare the intergroups. Cox regression was used for multivariable analysis. The confidence level was set at 95%, and *p* < 0.05 was considered statistically significant.

To address potential confounding and selection bias introduced by non-random group allocation, we performed multivariable Cox proportional hazards regression analysis. After adjusting for prespecified baseline covariates that were clinically relevant or showed imbalanced distribution between groups. These covariates included: tympanogram type at enrollment, preoperative pure-tone average, age, and AH ratio. The results are presented as adjusted hazard ratios (aHR) with 95% confidence intervals (CI).

Although this was a prospective cohort study where treatment allocation was based on parental preference rather than randomization, a post-hoc power analysis was conducted to determine the achieved statistical power of the primary comparison. The primary outcome for this analysis was the time to resolution of OME (as depicted in the Kaplan–Meier curves and analyzed by the log-rank test). Using the observed resolution rates, a total sample size of 73 patients (35 in the tympanocentesis group and 38 in the observation group) provided >80% power (*α* = 0.05, two-sided) to detect a hazard ratio (HR) of approximately 0.55 between the two groups, using G*Power software (version 3.1.9.7). This indicates that the study was adequately powered to detect a clinically significant difference in the time to healing.

## 3 Results

### 3.1 Baseline information

Seventy-three patients were included in this study, including 51 males and 22 females, aged 3–6 years, with a mean of (4.66 ± 1.22) years. Twenty children had one ear with OME (20 affected ears), including 11 and nine in the observation and tympanocentesis groups, respectively. Fifty-three children had OME bilaterally (106 affected ears), including 27 and 26 in the observation and tympanocentesis groups, respectively. Therefore, 126 affected ears were observed, with 61 ears in the tympanocentesis group and 65 in the observation group. All 61 ears in the tympanocentesis group were Type B tympanogram (100%), while among the 65 ears in the observation group, 47 were Type B (72.3%), and 18 were Type C (27.7%).

There were more type B ears in the tympanocentesis group, with a statistically significant difference (*χ*^2^ = 18.44, *p* < 0.01). The mean preoperative PTA was (22.35 ± 5.42) dB HL in the tympanocentesis group and (21.62 ± 5.92) dB HL in the observation group, without statistical difference in preoperative hearing level between the two groups (*p* = 0.549).

No significant differences were found in age, sex, or adenoid hypertrophy between the two groups (*p* > 0.05) (Table 1).

**Table 1:** Characteristics of the observation and tympanocentesis groups before surgery.

Baseline characteristic	Tympanocentesis [*n* = 35 (61 ears)]	Observation [*n* = 38 (65 ears)]	χ^2^	*P*
Age (years, ¯x± s)	5.00 ± 1.47	4.34 ± 0.81		0.074
Median	4.73	4.33		
Tympanogram [ears (%)]			18.44	<0.01
Type B	61 (100)	47 (72.3)		
Type C	0	18 (27.7)		
PTA (dB HL, ¯x±s)	22.35 ± 5.42	21.62 ± 5.92		0.549
AH (%)	0.88 ± 0.117	0.89 ± 0.102		0.960

### 3.2 Hearing outcomes over 12 months of follow-up

**Figure 1:**
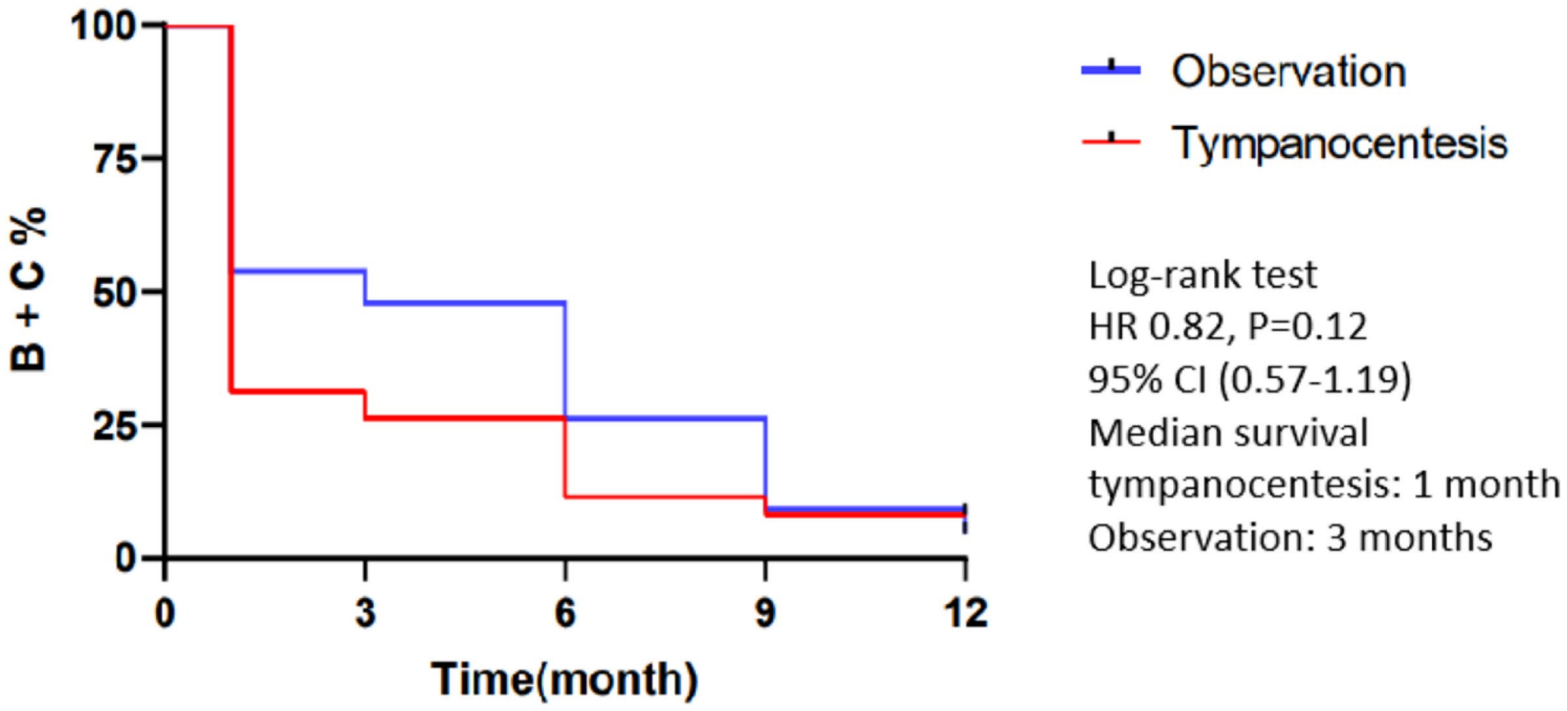
Kaplan–Meier curve of ears free from tympanogram type B/C during 12 months.

There were more children with type B tympanogram in the tympanocentesis group before the operation, but they turned into more types A and C at 1, 3, and 6 months after the operation (*p* < 0.05), suggesting the possibility that OME may resolve faster in ears that underwent tympanocentesis. At 9 and 12 months, all the ears in the two groups were free from type B tympanogram, and there were no statistically significant differences in tympanogram between the two groups, indicating that the long-term improvement of middle ear pressure was the same between the two treatments (Table 2). There were no differences between groups in the time to be free from types B and C tympanogram (HR = 0.82, *p* = 0.12) (Figure 1).

**Table 2:** Changes in tympanogram before and during 3 years after surgery.

Tympanogram	Percentage of ears (tympanocentesis/observation)						
	Before surgery	1 month after surgery	3 months after surgery	6 months after surgery	9 months after surgery	12 months after surgery	3 years after surgery
A	0.0%/0.0%	68.9%/46.2%	73.8%/52.3%	88.5%/73.8%	91.8%/90.8%	91.8%/95.4%	98.4%/96.9%
B	100.0%/72.3%	11.5%/24.6%	13.1%/15.4%	8.2%/3.1%	0.0%/0.0%	0.0%/0.0%	0.0%/0.0%
C	0.0%/27.7%	19.7%/29.2%	13.1%/32.3%	3.3%/20.0%	8.2%/9.2%	8.2%/4.6%	1.6%/3.1%
*P*	<0.01	0.03	0.023	0.011	>0.99	0.482	<0.01

**Figure 2:**
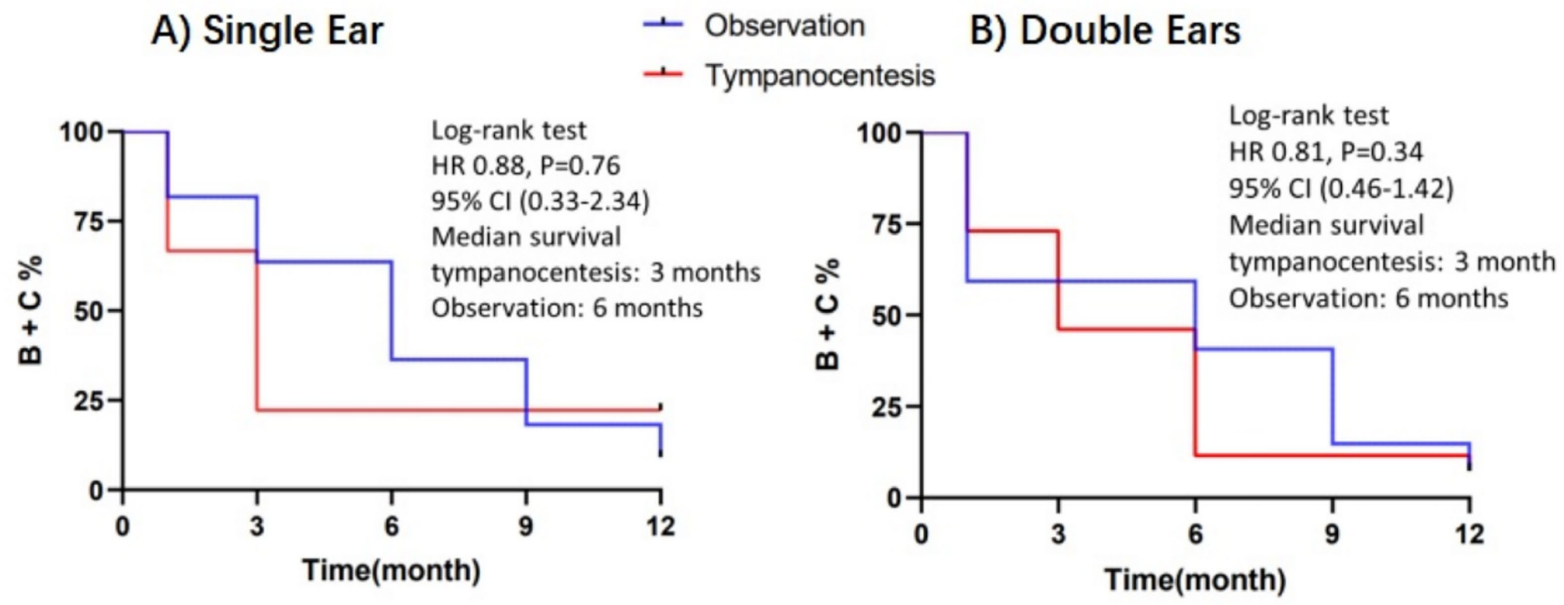
Kaplan-Meier curve of ears free from tympanogram type B/C during 12 months. **(A)** Patients with unilateral involvement; **(B)** Patients with bilateral involvement (both ears free from type B/C).

There were no statistically significant differences in PTA between the two groups overall. However, at 1 month postoperatively, the tympanocentesis group showed better hearing recovery than the observation group (*p* = 0.006). At 3 and 6 months postoperatively, no significant differences in hearing thresholds were observed between the two groups. By 9 and 12 months postoperatively, the tympanocentesis group had better PTA than the observation group (*p* < 0.05). Nevertheless, both groups had already recovered to normal hearing levels at these follow-up points (PTA ≤ 25 dB HL)(Table 3). The two groups had children with bilateral and unilateral involvement (Table 4). As mentioned above, the same treatment was used for both ears for children with bilateral involvement. OME was considered resolved only when both ears had recovered. Kaplan–Meier curve of ears free from types B and C tympanogram over the 12 months of follow-up is shown in Figure 2.

**Table 3:** Changes in PTA before and during 3 years after surgery.

PTA before and during 3 years after surgery							
PTA (dB HL, ‒x±s)	Before surgery	1 month after surgery	3 months after surgery	6 months after surgery	9 months after surgery	12 months after surgery	3 years after surgery
Tympanocentesis [*n* = 35 (61 ears)]	22.35 ± 5.42	12.55 ± 5.12	9.08 ± 7.01	6.93 ± 6.81	5.15 ± 4.42	3.55 ± 3.46	3.60 ± 3.54
Observation [*n* = 38 (65 ears)]	21.62 ± 5.92	13.67 ± 6.91	10.50 ± 6.23	7.79 ± 3.76	5.53 ± 3.13	4.04 ± 2.70	3.98 ± 2.77
*P*	0.549	0.006	0.057	0.112	0.000	0.025	0.001

**Table 4:** Changes in tympanogram of the unilateral or bilateral group before and during 3 years after surgery.

Time	Tympanogram	Unilateral group (*n* = 20)		Bilateral group (*n* = 53)	
		Observation	Tympanocentesis	Observation	Tympanocentesis
		(*n* = 11, 11 ears)	(*n* = 9, 9 ears)	(*n* = 27, 54 ears)	(*n* = 26, 52 ears)
Before surgery	B+C	11 (100.0%)	9 (100.0%)	54 (100.0%)	52 (100.0%)
	A	0 (0.0%)	0 (0.0%)	0 (0.0%)	0 (0.0%)
	B	9 (81.8%)	9 (100.0%)	38 (70.4%)	52 (100.0%)
	C	2 (18.2%)	0 (0.0%)	16 (29.6%)	0 (0.0%)
1 month after surgery	B+C	9 (81.8%)	6 (66.7%)	25 (46.3%)	30 (57.7%)
	A	2 (18.2%)	3 (33.3%)	29 (53.7%)	22 (42.3%)
	B	3 (27.3%)	4 (44.4%)	12 (22.2%)	9 (17.3%)
	C	6 (54.5%)	2 (22.2%)	13 (24.1%)	21 (40.4%)
3 months after surgery	B+C	7 (63.6%)	2 (22.2%)	24 (44.4%)	1 (1.9%)
	A	4 (36.4%)	7 (77.8%)	30 (55.6%)	38 (73.1%)
	B	3 (27.3%)	2 (22.2%)	7 (13.0%)	6 (11.5%)
	C	4 (36.4%)	0 (0.0%)	17 (31.5%)	8 (15.4%)
6 months after surgery	B+C	4 (36.4%)	2 (22.2%)	12 (22.2%)	5 (9.6%)
	A	7 (63.6%)	7 (77.8%)	41 (75.9%)	47 (90.4%)
	B	1 (9.1%)	1 (11.1%)	3 (5.6%)	4 (7.7%)
	C	3 (27.3%)	1 (11.1%)	10 (18.5%)	1 (1.9%)
9 months after surgery	B+C	2 (18.2%)	2 (22.2%)	4 (7.4%)	3 (5.8%)
	A	9 (81.8%)	7 (77.8%)	50 (92.6%)	49 (94.2%)
	B	0 (0.0%)	0 (0.0%)	0 (0.0%)	0 (0.0%)
	C	2 (18.2%)	2 (22.2%)	4 (7.4%)	3 (5.8%)
12 months after surgery	B+C	1 (9.1%)	2 (22.2%)	3 (5.6%)	3 (5.8%)
	A	10 (90.9%)	7 (77.8%)	52 (96.3%)	49 (94.2%)
	B	0 (0.0%)	0 (0.0%)	0 (0.0%)	0 (0.0%)
	C	1 (9.1%)	2 (22.2%)	2 (3.7%)	3 (5.8%)

### 3.3 Prognosis, recurrence and adverse effects

All 73 pediatric patients in this study completed all six postoperative follow-up visits.

At 1 month postoperatively, the tympanic membranes of the tympanocentesis group were all healed without otorrhea, tympanic membrane perforation, or infection. OME resolved in all 73 children within the first 12 months of follow-up. The mean recovery time of the tympanocentesis and observation groups was (2.46 ± 1.96) and (3.58 ± 2.65) months, respectively, and the median healing time was 1 month and 3 months (*p* = 0.10).

During the 1-year follow-up period, in the tympanocentesis group, OME persisted in one patient (two ears) and recurred after healing in two patients (four ears). In the observation group, acute otitis media occurred in one patient (two ears). Three of the four recurrences or new onset occurred in January.

Cox regression analysis showed no significant differences in the time to healing between the two groups (regression HR = 0.097, *p* = 0.936). After adjusting for baseline tympanogram type, PTA, age, and AH ratio in a multivariable model, the effect of the tympanocentesis group remained non-significant (adjusted HR = 0.665, 95% CI 0.44 to 1.00, *p* = 0.054). None of the other covariates were significant independent predictors.

Furthermore, at 3 years postoperatively, PTA returned to normal levels in patients in both groups, and no history of OME lasting more than 3 months occurred during the 3–5 years postoperative period (Table 4).

### 3.4 Quality of life

The OM-6 questionnaire revealed no significant differences in overall quality of life between the tympanocentesis and observation groups at 1 month, 1 year, and 3 years postoperatively. Both groups displayed a downward trend in scores over time, indicating improvement. Notably, regarding “Caregiver concerns,” the tympanocentesis group performed significantly better than the observation group at 1 month post-surgery (*p* = 0.004) (Table 5). In contrast, the observation group surpassed the tympanocentesis group at 1-year post-surgery (*p* = 0.046).

**Table 5:** Comparison of the quality of life of OM-6 questionnaire in children after operation in two groups.

OM-6 questionnaire during 3 years after surgery								
Time	Group	Physical suffering	Hearing loss	Speech impairment	Emotional distress	Activity limitations	Caregiver concerns	Overall score
1 month after surgery	Tympanocentesis	2.97 ± 0.92	1.66 ± 0.68	3.60 ± 0.81	2.09 ± 0.66	4.54 ± 1.67	5.68 ± 0.87	20.54 ± 2.22
	Observation	3.79 ± 1.32	2.55 ± 0.83	4.21 ± 1.49	2.21 ± 1.07	3.55 ± 0.86	6.08 ± 0.71	22.39 ± 2.92
	P	0.595	0.776	0.181	0.216	0.515	0.004**	0.935
12 months after surgery	Tympanocentesis	1.20 ± 0.41	1.40 ± 0.50	1.26 ± 0.44	1.46 ± 0.51	2.54 ± 0.56	3.81 ± 0.66	11.69 ± 1.08
	Observation	1.53 ± 0.51	1.58 ± 0.50	1.39 ± 0.68	1.37 ± 0.59	1.21 ± 0.41	3.50 ± 0.65	10.58 ± 1.11
	P	1.000	0.267	0.669	0.557	0.730	0.046*	0.673
3 years after surgery	Tympanocentesis	1.46 ± 0.70	1.60 ± 0.60	1.66 ± 0.64	1.46 ± 0.66	2.89 ± 0.72	3.29 ± 1.25	12.34 ± 1.55
	Observation	1.45 ± 0.89	1.55 ± 1.08	1.21 ± 0.41	1.42 ± 0.50	1.37 ± 0.49	2.95 ± 1.01	9.95 ± 2.78
	*P*	0.776	0.170	0.651	0.528	0.073	0.735	0.636

*p < 0.05; **p < 0.01.

## 4 Discussion

OSA and OME are two common conditions in children. Adenotonsillar hypertrophy is the most common cause of OSA, and adenotonsillectomy continues to be the primary treatment for this issue (6). About 90% of children have OME before school age (7), and they develop, on average, four episodes of OME every year (8). Most episodes of OME resolve spontaneously within 3 months, but 30–40% of children have repetitive OME episodes (9).

According to published guidelines, clinicians should not routinely screen children for OME if they are not at risk and do not have possible symptoms of OME (5). The most common symptoms of middle ear effusion are hearing difficulties and ear discomfort, and some children may present poor school performance, behavioral problems, balance (vestibular) problems, sleep disturbance, and emotional distress. These symptoms are similar to childhood OSA. Still, the two conditions can coexist, and children with OSA are prone to OME. Indeed, Sogebi et al. reported that 29.2% of children with adenoid enlargement had asymptomatic OME, and the factors associated with OME were increased age and weight of the patients (4). Khadgi et al. showed that adenoid hypertrophy positively correlates with conductive hearing loss in pediatric patients and suggested that proper screening and early management should be done to prevent hearing loss in children (10). Similar findings have been reported by Suresh et al., i.e., that larger adenoids were associated with lower middle ear pressure and reduced compliance and that a significant majority of patients with enlarged adenoids also had OME (11). Liu et al. also proposed conducting routine audiological examinations for all children with OSA (12). Considering the age, poor expression, or mild symptoms not observed by guardians, it is probably necessary to screen perioperatively for children with OME and OSA.

The updated NICE guidance made strong recommendations that clinicians should manage children with OME at low risk of complications with observation for 3 months from the date of effusion onset (if known) or 3 months from the date of diagnosis (if onset is unknown) (13). The guidance also recommends against using intranasal or systemic steroids, systemic antibiotics, antihistamines, or decongestants for treating OME. On the other hand, clinicians should obtain an age-appropriate hearing test if OME persists for 3 months. If hearing loss is identified, surgery should be performed for OME, including tympanostomy tubes, adenoidectomy, or both (5). However, at least 25% of OME episodes persist for 3 months (13).

The situation faced by this study is a clinical dilemma ignored by the current guidelines, i.e., children found to have adenotonsillar hypertrophy due to OSA and OME on preoperative examination, but OME is asymptomatic. OME for over 3 months cannot be closely observed when adenoidectomy is necessary (14). There is a lack of relevant clinical studies, although adenotonsillar hypertrophy with OME in children has been extensively studied (12, 15–17). Table 6 presents studies on the efficacy of myringotomy with or without adenoidectomy in treating OME, and the practice guidelines were updated by incorporating some of the findings. According to the current view, tympanostomy alone has no long-term benefits for OME, while adenoidectomy can reduce the risk of second tympanostomy tube insertion. Compared with children in these studies who have been watched with OME for > 3 months, the children with asymptomatic OME in the present study were managed with a more conservative regimen. The parents must be given clinically informed recommendations in this situation because children with OSA require general anesthesia for adenotonsillectomy, including whether OME needs to be managed and how it should be managed.

**Table 6:** Summary of the clinical characteristics of six OME studies.

Author/year	Study Design	Chief Complaint	Ears	Age/year	F/U Period/month	Surgical procedures	Pre-surgery Hearing/dB	Post-surgery Hearing/dB	Postoperative Outcomes	Recurrence/%
Abdul-Baqi et al., 2001 (19)	Prospective	Hearing impairment, snoring	48 (2 lost to F/U)	5.7 (2-14)	6	A	/	/	/	17.40%
			48	5.9 (3-13)		AS	/	/	/	4.20%
Popova et al., 2010 (20)	Prospective	Nasal obstruction	72	5.1(3.8-6.3)	18	A+M	32.3	13.9^a^, 7.6^c^, 5.5^d^	/	14.00%
			84	5(3.5-7.2)		A+T	31.4	14.1^a^, 8.0^c^, 6.3^d^	10 patients, one otorrhea episode, 5 patients, two, 1 patient, three, 1 patient, four or more; 7 patients, tube occlusions	10.00%
Vlastos et al., 2011 (17)	Prospective	Snoring	27	4.4 (3-7)	12	A+M	32.7	28.88^c^, 25.47^d^	/	20.00%
			25	4.6 (3-7)		A+T	31.2	23.71^c^, 23.19^d^	/	0.00%
Rasheed et al., 2016 (21)	Prospective	Nasal obstruction, snoring, hearing impairment	62	6.1 (5-7)	6	A+M	27.9	19.4^b^,13.6^c^	/	/
			64	5.6 (5-7)		A+T	28.3	13.2^b^, 6.8^c^	/	/
Tao et al., 2020 (18)	Prospective	Snoring, hearing impairment	124	7.2 (4-12)	12	A+M	28.6	/	/	20.2%^b^, 6.5%^c^, 5.6%^d^
			134	7.0 (4-12)		A+T	26.7	/	26 ears, otorrhea, 4 ears, perforation, 11 ears, blocked tympanic membrane vent, 6 ears, tympanic membrane calcification	1%^a^, 7.5%^b^, 6.7%^d^
Rasheed et al., 2023 (22)	Prospective	Nasal obstruction, snoring, hearing impairment	24	8.11 (6-12)	3	A+M	27.3	18.2^a^, 14.8^b^	/	/
			10			AS+M			/	/
			26	8.87 (6-12)		A+T	29.5	10.6^a^, 3.5^b^	/	/
			6			AS+T			/	/

F/U, follow-up; A, adenoidectomy; AS, adenotonsillectomy; M, myringotomy; T, tympanostomy.

^a^=1 month, ^b^=3 months, ^c^=6 months, ^d^=12 months.

For OME, the disease state of concern is not the asymptomatic presence of fluid but previously undetected hearing loss or other OME-induced symptoms that would benefit from treatment. Early intervention is mainly valuable in preventing or managing hearing loss. Tympanostomy tube insertion is one of the common surgical methods for treating OME. Compared with simple myringotomy, it confers a short-term benefit for OSA syndrome (18). In terms of hearing loss, it can bring short-term improvement but has no impact on long-term hearing (18–21). However, in treating recurrent OME, tympanostomy tube insertion can effectively shorten the time of middle ear effusion and significantly reduce the recurrence rate (22). For children undergoing surgical treatment during autumn and very young children, tympanostomy tube insertion is usually recommended (17). It should be noted that tube insertion can cause a series of complications, with the most common tube-related sequelae being otorrhea, which is seen in approximately 16% of children within 4 weeks of surgery and 26% of children at any time when the tube remains in place (mean, 12–14 months) (18, 23). Complications include an obstructed tube lumen in 7% of intubated ears, premature extrusion of the tube in 4%, and tube displacement into the middle ear in 0.5%. Longer-term sequelae of tympanostomy tubes include visible changes in the appearance of the tympanic membrane (e.g., atrophy, retraction, perforation, and myringosclerosis). The post-tympanostomy tube sequela most likely to require intervention is persistent perforation, which occurs in about 2 to 3% of children (5). About one in every four children may need surgery again to replace falling-out tubes. Baths, swimming, and air travel are fine, but some children need earplugs if dirty water bothers their ears when bathing, swimming, or diving. The insertion of tympanostomy tubes is definitely out of indication for asymptomatic OME and may be considered overtreatment. On the other hand, if only tympanocentesis or observation is performed, the child and parents may face general anesthesia again for tympanostomy tubes when the middle ear effusion does not resolve, and hearing loss occurs after 3 months.

The clinician should determine if there are risk factors that would predispose the children to undesirable sequelae or predict the persistence of the effusion to be able to make the most optimal treatment selection. The longer the effusion is present, the more the resolution rate decreases, and relapse becomes more common (24). The risk factors associated with a reduced likelihood of spontaneous resolution of OME include the onset of OME in the summer or fall season, hearing loss >30 dB HL in the better-hearing ear, history of prior tympanostomy tubes, and not having a prior adenoidectomy (25). Since adenoid hypertrophy is a common cause of OSA and OME in children, adenoidectomy may improve OME. Adenoidectomy alone offers comparable rates of OME control compared with tympanostomy tubes at 6 and 12 months (26) but may have a less reliable impact in the short term. Adenoidectomy also reduces the need for repeat surgery of ear tubes by about 50% (27).

In addition, a conventional myringotomy, alone or in association with other therapies, is not considered a possible approach to OME because it heals after 10 days, which is usually too short for achieving a therapeutic effect (28), but myringotomy and aspiration may induce a temporary threshold shift in auditory brainstem response (29). A 3-week period of trans-tympanic ventilation is considered adequate for complete middle ear fluid elimination. However, when a child has just undergone adenoidectomy, postoperative swelling of structures such as the torus tubarius may lead to eustachian tube ostium obstruction, and the possibility of an increased risk of retrograde infection cannot be ignored.

In the present study, hearing improvement was achieved immediately after surgery in the tympanocentesis group, with a significantly higher level of hearing and effusion improvement 1 month after surgery than the observation, even though OME was more serious. The mean duration of the disease in the tympanocentesis group was shorter than in the observation group, but the multivariable time-dependent Cox regression analysis showed no significant differences in the time to healing between the two groups, which is similar to the results of a previous meta-analysis (30). During the 1-year follow-up, in the tympanocentesis group, OME persisted in one patient (two ears) and recurred after healing in two patients (four ears), while in the observation group, acute otitis media occurred in one patient (two ears) after being cured. Of note, three of these four children had a recurrence or new onset in January. Indeed, winter is prone to recurrent OME or AME. One year after surgery, both groups returned to a normal hearing level. In addition, all children recovered well from tonsils and adenoid surgery.

The immediate and sustained improvements in the tympanocentesis group and the shorter recovery time may suggest that tympanocentesis is effective in treating OME in children with OSA. The findings suggested that children who underwent tympanocentesis experienced an immediate improvement in tympanogram, as supported by better PTA at 1 month postoperatively in the tympanocentesis group than in the observation group, but the difference disappeared at 3 and 6 months, and the two groups had PTA in the normal range at 9 and 12 months. As hearing difficulties are one of the primary symptoms associated with OME, immediate recovery of OME can positively impact a child’s overall well-being. It may address symptoms like poor school performance, behavioral problems, and emotional distress related to hearing difficulties (31). Nevertheless, the Cox regression analysis supports observation. Within 12 months after OME onset, tympanogram suggested that observation played equally important roles in recovering childhood OSA with asymptomatic OME than tympanocentesis. The results implied that selecting either method may yield comparable outcomes. Nevertheless, it must be mentioned that the patients with PTA ≥ 35 dB and clinical symptoms or specific indications for tympanostomy and tube placement were excluded from the present study. Therefore, the patients should be carefully selected.

The penultimate aim of this study is to prevent unnecessary testing, subsequent visits, parental or child anxiety, and medical expenses for children with OSA and asymptomatic OME that can be self-limited after adenoidectomy and tonsillectomy (partial or total). Recent guidelines focus on efficacy prediction, that is, to reduce the recurrence rate of OME and the incidence of AME. In this study, adjacent patterns were observed between the adenoid and torus tubarius. Compared with the effect of the adenoid-to-choana relationship on OSA, whether the adenoid-to-torus tubarius relationship impacts the severity and duration of OME still requires prognosis evidence and deserves further study. Nevertheless, the parents should be engaged in the preoperative discussion to carefully weigh the benefits and possible complications (32). Furthermore, tympanocentesis may lead to a shorter duration of OME and prevent unnecessary testing, subsequent visits, and parental or child anxiety. It remains a simple, optimized, economical, and effective treatment strategy.

Children with OSA and OME, even when OME is asymptomatic, may experience measurable impacts on quality of life since the interplay between these conditions can affect sleep, behavior, emotional functioning, and overall well-being (33). Parental reports indicate high rates of sleep disruption (64%), behavioral problems (49%), and speech or hearing concerns (35–62%) in children with OME, and the burden of OME extends to caregivers, who report increased stress and concerns about their child’s development and daily functioning (33, 34). Children with OME often exhibit significant symptoms of OSA, including sleep disturbances and associated impairment in quality of life, and the OSA-18 questionnaire, a tool for assessing sleep-related quality of life, shows that a substantial proportion of children with OME have moderate to severe OSA symptoms, further diminishing their quality of life (35, 36). Even when OME is asymptomatic, it can still contribute to subtle but significant reductions in QoL, especially when coupled with OSA (36). Although the results showed a similar overall quality of life between the two groups and similar improvement over time, the tympanocentesis group showed better scores in the “caregiver concern,” supporting the above notion that tympanocentesis may help manage parental anxiety. Additionally, at 1 month post-surgery, except for the “Activity limitations,” the tympanocentesis group’s scores were lower than those of the observation group. It can be concluded that tympanocentesis therapy for asymptomatic OME can alleviate parents’ anxiety about their child’s condition in the short term and improve the child’s quality of life. However, due to the presence of a medically created perforation in the eardrum, there may be some impact on the child’s daily activities such as bathing and going out, and this impact may persist for an extended period. At 1 year postoperatively, parents of children in both groups still exhibited a certain degree of disease-related anxiety (with average scores in both groups exceeding 3.5), but the mean scores for actual behavioral observations in children were below 2, suggesting that the children’s quality of life has improved. Our study can be included into “shared decision-making tools” for the treatments of OSA in children. A multidisciplinary approach with shared decision making can reduce decisional conflict and might improve compliance for therapies that require long-term adherence for effectiveness (37).

## 5 Limitations of the study

The limitation of this study is that the seasonal factor was not considered when designing the experiment, which may have affected the outcome. The season of operation can also affect the recurrence of OME, complications, tympanic membrane prognosis, and hearing recovery (14). Furthermore, more flexible treatment options can be adopted based on the duration and nature of middle ear effusion. The severity classification of OSA is based on the PSG index, and OME may be related to the index. In the present study, the grouping based on parents’ choice of treatment was not completely random. Although we repeatedly emphasized that hearing improvement was achieved a higher level in the tympanocentesis group 1 month after surgery, even if there was a higher proportion of type B tympanogram before the operation, it still might have a confounding early outcomes. In addition, among the four recurrences, three were in thetympanocentesis group, suggesting that we need to analyze any baseline factor deeply. Fourth, the sample size, while adequate for describing outcomes in this real-world cohort, may have been insufficient to detect a small but clinically meaningful difference in OME resolution time between the two strategies. The observed hazard ratio of 0.665 (95% CI 0.44 to 1.00, *p* = 0.054) indicates a trend favoring tympanocentesis but with considerable uncertainty, as reflected by the wide confidence interval crossing unity. Future studies with larger sample sizes are warranted to provide a more precise estimate of the treatment effect. In future studies, a more comprehensive experimental design should be considered.

## 6 Conclusion

Tympanocentesis and observation demonstrated efficacy in pediatric patients with OSA and asymptomatic OME over the 3-year follow-up period, with minimal recurrence. Tympanocentesis may lead to a shorter duration of OME, offering a simple, optimized, economical, and effective treatment strategy. The findings emphasize the importance of individualized treatment decisions to address each patient’s unique needs.

## Data Availability

The original contributions presented in the study are included in the article/supplementary material, further inquiries can be directed to the corresponding author.

## References

[1] Mitchell RB Pereira KD Friedman NR. Sleep-disordered breathing in children: survey of current practice. Laryngoscope. (2006) 116:956–8. doi: 10.1097/01.MLG.0000216413.22408.FD, 16735907

[2] Marcus CL Moore RH Rosen CL Giordani B Garetz SL Taylor HG . A randomized trial of adenotonsillectomy for childhood sleep apnea. N Engl J Med. (2013) 368:2366–76. doi: 10.1056/NEJMoa1215881, 23692173 PMC3756808

[3] Soh HJ Rowe K Davey MJ Horne RSC Nixon GM. The OSA-5: development and validation of a brief questionnaire screening tool for obstructive sleep apnea in children. Int J Pediatr Otorhinolaryngol. (2018) 113:62–6. doi: 10.1016/j.ijporl.2018.07.029, 30174012

[4] Sogebi OA Oyewole EA Ogunbanwo O. Asymptomatic otitis media with effusion in children with adenoid enlargement. J Natl Med Assoc. (2021) 113:158–64. doi: 10.1016/j.jnma.2020.08.005, 32838976

[5] Rosenfeld RM Tunkel DE Schwartz SR Anne S Bishop CE Chelius DC . Executive summary of clinical practice guideline on tympanostomy tubes in children (update). Otolaryngol Head Neck Surg. (2022) 166:189–206. doi: 10.1177/01945998211065661, 35138976

[6] Marcus CL Brooks LJ Draper KA Gozal D Halbower AC Jones J . Diagnosis and management of childhood obstructive sleep apnea syndrome. Pediatrics. (2012) 130:e714–55. doi: 10.1542/peds.2012-1671 22926176

[7] Simon F Haggard M Rosenfeld RM Jia H Peer S Calmels MN . International consensus (ICON) on management of otitis media with effusion in children. Eur Ann Otorhinolaryngol Head Neck Dis. (2018) 135:S33–9. doi: 10.1016/j.anorl.2017.11.009, 29398506

[8] Mandel EM Doyle WJ Winther B Alper CM. The incidence, prevalence and burden of OM in unselected children aged 1-8 years followed by weekly otoscopy through the “common cold” season. Int J Pediatr Otorhinolaryngol. (2008) 72:491–9. doi: 10.1016/j.ijporl.2007.12.008, 18272237 PMC2292124

[9] Se S Ao B S B. Otitis Media with Effusion in Young Children: Clinical Practice Guideline No. 12. Rockville, MD: Agency for Healthcare Research and Quality (1994).

[10] Khadgi A Koirala K Maharjan S Chalise K Dhungana I Babu KB. Correlation of conductive hearing impairment with sizes of adenoids in the pediatric age group: an observational case-control study. Cureus. (2023) 15:e44439. doi: 10.7759/cureus.44439, 37791228 PMC10544040

[11] Suresh A Mahajan G Thomas J Babu M. Correlation of the size of adenoids with impedance audiometry findings. Cureus. (2024) 16:e62453. doi: 10.7759/cureus.62453, 39015869 PMC11250518

[12] Liu CB Shi YH Li XY Fan ZT. Prevalence and risk factors of otitis media with effusion in children with obstructive sleep apnea. Eur Rev Med Pharmacol Sci. (2023) 27:5445–52. doi: 10.26355/eurrev_202306_32780, 37401280

[13] Connolly R Paing A Reeves T Joshi D Kennedy V Royds J. Otitis media with effusion in under 12s: summary of updated NICE guidance. BMJ. (2023) 383:2314. doi: 10.1136/bmj.p2314, 37945047

[14] Ahmad Z Krüger K Lautermann J Lippert B Tenenbaum T Tigges M . Adenoid hypertrophy- diagnosis and treatment: the new S2k guideline. HNO. (2023) 71:67–72. doi: 10.1007/s00106-023-01299-6, 37491540 PMC10409824

[15] Rosenfeld RM Kay D. Natural history of untreated otitis media. Laryngoscope. (2003) 113:1645–57. doi: 10.1097/00005537-200310000-00004, 14520089

[16] Chen W Yin G Chen Y Wang L Wang Y Zhao C . Analysis of factors that influence the occurrence of otitis media with effusion in pediatric patients with adenoid hypertrophy. Front Pediatr. (2023) 11:1098067. doi: 10.3389/fped.2023.1098067, 36911018 PMC9992982

[17] Vlastos IM Houlakis M Kandiloros D Manolopoulos L Ferekidis E Yiotakis I. Adenoidectomy plus tympanostomy tube insertion versus adenoidectomy plus myringotomy in children with obstructive sleep apnoea syndrome. J Laryngol Otol. (2011) 125:274–8. doi: 10.1017/S0022215110002549, 21205368

[18] Tao J Luo R Chen Y Hou C Qin H. Myringotomy or tympanostomy tube insertion, comparison of surgical treatment of adenoid hypertrophy and otitis media with effusion in children. J Clin Otorhinolaryngol Head Neck Surg. (2020) 34:207–10. doi: 10.13201/j.issn.2096-7993.2020.03.005, 32791583 PMC10127848

[19] Abdul-Baqi KJ Shakhatreh FM Khader QA. Use of adenoidectomy and adenotonsillectomy in children with otitis media with effusion. Ear Nose Throat J. (2001) 80:647–50. doi: 10.1177/014556130108000910, 11579851

[20] Popova D Varbanova S Popov TM. Comparison between myringotomy and tympanostomy tubes in combination with adenoidectomy in 3-7-year-old children with otitis media with effusion. Int J Pediatr Otorhinolaryngol. (2010) 74:777–80. doi: 10.1016/j.ijporl.2010.03.054, 20399511

[21] Rasheed AM. Adenoidectomy with Myringotomy and Tympanostomy Tube Versus Adenoidectomy with Myringotomy in Treatment of Otitis Media with Effusion in 5-7 Years Old Children. Al-Kindy Col. Med. J. (2016) 12:83–86. Available online at: https://jkmc.uobaghdad.edu.iq/index.php/MEDICAL/article/view/349 (Accessed April 14, 2026).

[22] Rasheed AM Abbas AM Hilal SA Homadi NJ. Adenoidectomy and endoscopic myringotomy with and without ventilation tube insertion for treatment of otitis media with effusion in 6-12 years old children. Int Tinnitus J. (2023) 27:27–33. doi: 10.5935/0946-5448.20230005, 38050881

[23] Kay DJ Nelson M Rosenfeld RM. Meta-analysis of tympanostomy tube sequelae. Otolaryngol Head Neck Surg. (2001) 124:374–80. doi: 10.1067/mhn.2001.113941, 11283489

[24] van Balen FA de Melker RA. Persistent otitis media with effusion: can it be predicted? A family practice follow-up study in children aged 6 months to 6 years. J Fam Pract. (2000) 49:605–11. 10923569

[25] Medical Research Council Multicentre Otitis Media Study Group. Surgery for persistent otitis media with effusion: generalizability of results from the UK trial (TARGET). Trial of alternative regimens in glue ear treatment. Clin Otolaryngol Allied Sci. (2001) 26:417–24. doi: 10.1046/j.1365-2273.2001.00495.x, 11678951

[26] Wallace IF Berkman ND Lohr KN Harrison MF Kimple AJ Steiner MJ. Surgical treatments for otitis media with effusion: a systematic review. Pediatrics. (2014) 133:296–311. doi: 10.1542/peds.2013-3228, 24394689

[27] Mikals SJ Brigger MT. Adenoidectomy as an adjuvant to primary tympanostomy tube placement: a systematic review and meta-analysis. JAMA Otolaryngol Head Neck Surg. (2014) 140:95–101. doi: 10.1001/jamaoto.2013.5842 24287958

[28] Midgley EJ Dewey C Pryce K Maw AR. The frequency of otitis media with effusion in British pre-school children: a guide for treatment. ALSPAC Study Team. Clin Otolaryngol Allied Sci. (2000) 25:485–91. doi: 10.1046/j.1365-2273.2000.00360.x 11122285

[29] Dietrich M Schade H Nadal J Keiner S Schade G. Clinical decision making for intraoperative auditory brainstem response testing in children following Tympanostomy tube placement. J Clin Med. (2023) 12:830. doi: 10.3390/jcm12030830, 36769478 PMC9917660

[30] Lous J Burton MJ Felding JU Ovesen T Rovers MM Williamson I. Grommets (ventilation tubes) for hearing loss associated with otitis media with effusion in children. Cochrane Database Syst Rev. (2005) 1:Cd001801. doi: 10.1002/14651858.CD001801.pub3 15674886

[31] Vercammen C Ferguson M Kramer S. Well-hearing is well-being. Hear Rev. (2020) 27:18–22.

[32] Akanmode AM Winters R. "Tympanocentesis". In: StatPearls [Internet]. Treasure Island (FL): StatPearls Publishing (2026) 32809429

[33] Homøe P Heidemann CH Damoiseaux RA Lailach S Lieu JEC Phillips JS . Panel 5: impact of otitis media on quality of life and development. Int J Pediatr Otorhinolaryngol. (2020) 130:109837. doi: 10.1016/j.ijporl.2019.109837 31883704 PMC7197055

[34] Bergeron M Duggins AL Cohen AP Leader BA Ishman SL. The impact of persistent pediatric obstructive sleep apnea on the quality of life of patients' families. Int J Pediatr Otorhinolaryngol. (2020) 129:109723. doi: 10.1016/j.ijporl.2019.109723, 31678898

[35] Huang CC Wu PW Chiu CH Lee TJ Chen CL. Assessment of sleep-disordered breathing in pediatric otitis media with effusion. Pediatr Neonatol. (2022) 63:25–32. doi: 10.1016/j.pedneo.2021.06.013, 34391662

[36] Buckingham B. Sleep disordered breathing with otitis media with effusion in children. Pediatr Ther. (2022) 12:449. doi: 10.35841/2161-0665.22.12.449

[37] Ersu R Chen ML Ehsan Z Ishman SL Redline S Narang I . Persistent obstructive sleep apnoea in children: treatment options and management considerations. Lancet Respir Med. (2022) 11:283–96. doi: 10.1016/S2213-2600(22)00262-4 36162413

